# Bacterial Translocation as Inflammatory Driver in Crohn’s Disease

**DOI:** 10.3389/fcell.2021.703310

**Published:** 2021-09-07

**Authors:** Raquel Linares, Rubén Francés, Ana Gutiérrez, Oriol Juanola

**Affiliations:** ^1^Hepatic and Intestinal Immunobiology Group, Department of Clinical Medicine, Miguel Hernández University, San Juan de Alicante, Spain; ^2^CIBERehd, Instituto de Salud Carlos III, Madrid, Spain; ^3^Instituto ISABIAL, Hospital General Universitario de Alicante, Alicante, Spain; ^4^Servicio de Medicina Digestiva, Hospital General Universitario de Alicante, Alicante, Spain; ^5^Translational Research Laboratory, Gastroenterology and Hepatology, Ente Ospedaliero Cantonale, Lugano, Switzerland; ^6^Faculty of Biomedical Sciences, Universitá della Svizzera Italiana, Lugano, Switzerland

**Keywords:** Crohn’s disease, bacterial translocation, intestinal permeability, dysbiosis, NOD2, inflammatory response, anti-TNF-α

## Abstract

Crohn’s disease (CD) is a chronic inflammatory disorder of the gastrointestinal tract responsible for intestinal lesions. The multifactorial etiology attributed to CD includes a combination of environmental and host susceptibility factors, which result in an impaired host–microbe gut interaction. Bacterial overgrowth and dysbiosis, increased intestinal barrier permeability, and altered inflammatory responses in patients with CD have been described in the past. Those events explain the pathogenesis of luminal translocation of bacteria or its products into the blood, a frequent event in CD, which, in turn, favors a sustained inflammatory response in these patients. In this review, we navigate through the interaction between bacterial antigen translocation, permeability of the intestinal barrier, immunologic response of the host, and genetic predisposition as a combined effect on the inflammatory response observed in CD. Several lines of evidence support that translocation of bacterial products leads to uncontrolled inflammation in CD patients, and as a matter of fact, the presence of gut bacterial genomic fragments at a systemic level constitutes a marker for increased risk of relapse among CD patients. Also, the significant percentage of CD patients who lose response to biologic therapies may be influenced by the translocation of bacterial products, which are well-known drivers of proinflammatory cytokine production by host immune cells. Further mechanistic studies evaluating cellular and humoral immune responses, gut microbiota alterations, and genetic predisposition will help clinicians to better control and personalize the management of CD patients in the future.

## Introduction

Crohn’s disease (CD) is a type of chronic idiopathic inflammatory bowel disease (IBD) that may affect any part of the gastrointestinal (GI) tract and causes inflammatory, stricturing, or penetrating intestinal lesions (Torres et al., 2017). The prevalence of CD has increased worldwide in the past 50 years, and it imposes a considerable economic burden on health systems as it requires new and costly treatment options and trained specialists to manage CD patients (Ng et al., 2017; Windsor and Kaplan, 2019). The etiology of CD includes several aspects involving environmental factors, genetic susceptibility, and the impaired immune interaction of the host with the intestinal microbiota (Chang, 2020).

The immune response in CD is induced by different cell types such as neutrophils and macrophages that act together with epithelial cells in promoting and generating inflammatory phenomena by releasing soluble factors as tumor necrosis factor-alpha (TNF-α) and antimicrobial peptides like defensins and cathelicidins (Ramasundara et al., 2009; Gutiérrez et al., 2011). Intestinal bacteria are key contributors to the onset, perpetuation, and pathogenesis of chronic intestinal inflammation suggesting a disturbed immune gut response to bacterial antigens. This hypothesis is supported by several lines of evidence: (1) CD clinical lesions are mainly located in the distal ileum and colon, which are regions of high microbial concentration, (2) several studies demonstrate that luminal bacteria are necessary for the development of disease in murine models (Harper et al., 1985; Elson et al., 2005), and (3) CD patients present dysbiosis or reduced biodiversity in the composition of their gut microbiota associated with increased bacteria with proinflammatory properties and less anti-inflammatory bacterial species (Willing et al., 2010; Chassaing and Darfeuille-Michaud, 2011; Manichanh et al., 2012).

Our gut epithelial cells act as a physical barrier between the lumen of the GI tract and the inner mucosa contributing to nutrient and fluid absorption and impeding intact bacteria penetration. Genetic polymorphisms affecting barrier function or chronic inflammation may contribute to an abnormal intestinal permeability and, therefore, favor bacterial translocation (BT) and aggravate the course of disease. Genes associated with intestinal homeostasis involve autophagy, innate and adaptive immune regulation, microbial defense, or barrier function, among others (Franke et al., 2010; Khor et al., 2011). Some risk loci might influence immunological function such as nucleotide-binding oligomerization domain-containing 2 (NOD2), which encodes an intracellular receptor for muramyl dipeptide (MDP), a component in bacterial cell walls (Inohara et al., 2003). In this regard, three common variants of NOD2 loci apparently confer susceptibility to CD (Hugot et al., 2001) suggesting that an impaired response to bacterial antigens may contribute, and further studies indicate that low Foxp3+ regulatory T-cell (Treg) counts and a variant NOD2 genotype can be markers of loss of response to anti-TNF-α in CD patients (Juanola et al., 2014).

In this review, we integrate the effect of bacterial antigen translocation, the host immunologic response, and the genetic background in the inflammatory response observed in CD.

## Gut-Derived Bacterial Antigen Translocation

Bacterial translocation is known as the passage of bacteria or its products, such as endotoxins, from the GI tract to mesenteric lymph nodes and systemic circulation (Alexander et al., 1990). This event has been demonstrated in CD by several studies in which the presence of bacteria in the lymph nodes (Takesue et al., 2002; Peyrin-Biroulet et al., 2012; O’Brien et al., 2014) or bacterial genomic fragments in the blood (Gutiérrez et al., 2009, 2011, 2014) are detected. Bacterial passage from the bowel lumen is a common phenomenon in CD, and it is involved in the pathogenesis by inducing, perpetuating, or exacerbating an inflammatory state (Swank and Deitch, 1996). The risk factors influencing BT are mainly intestinal bacterial overgrowth or dysbiosis, increased intestinal permeability, and local and systemic immunological alterations and can be followed in Table 1.

**TABLE 1:**
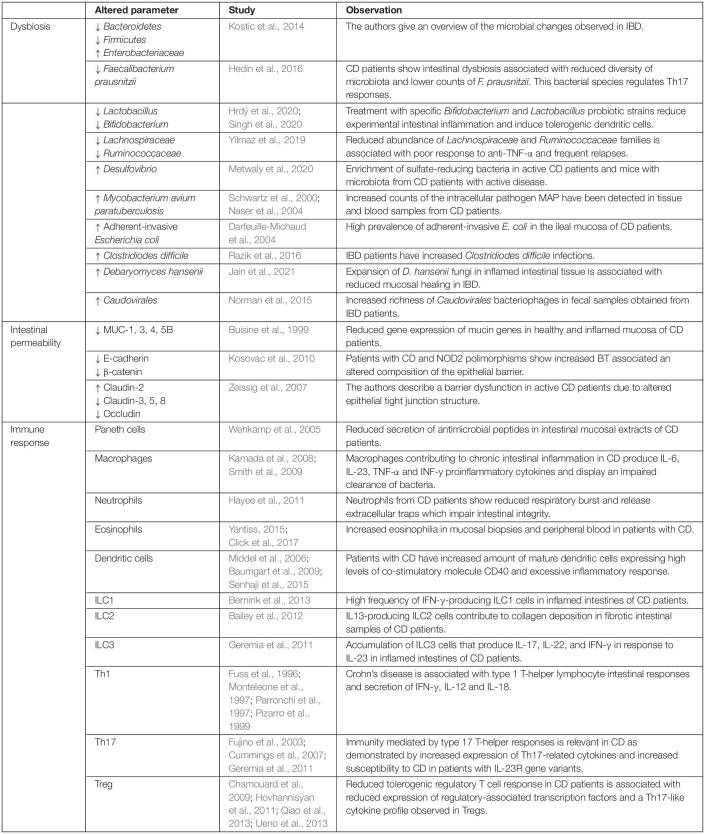
Risk factors for bacterial translocation in Crohn’s disease include intestinal dysbiosis, altered intestinal integrity, and immune dysfunction.

Human species have evolved with the symbiotic intestinal microbiota, which is composed of 10 (Franke et al., 2010) to 10 (Inohara et al., 2003) microorganisms including bacteria, fungus, archaea, and viruses whose total genome represents 100 times our own genome (Gill et al., 2006). GI microbiota is mainly composed of four bacterial divisions: *Firmicutes*, *Bacteroidetes*, *Proteobacteria*, and *Actinobacteria*, but the composition and the luminal concentrations of bacterial species may vary in the different GI tract regions (Eckburg et al., 2005). These commensal bacteria provide an abundant source of antigens that can activate pathogenic immune responses resulting in chronic intestinal inflammation and clinical manifestations of CD-susceptible patients.

An increase in the number and/or altered composition of microbial species in the small bowel is known as small intestinal bacterial overgrowth (SIBO), which is potentially caused by fistulae, strictures, a slowed intestinal transit, low acid gastric secretion, and altered local immune activity such as common variable immunodeficiencies associated with low IgA (Pignata et al., 1990; Husebye, 2005; Klaus et al., 2009). Patients with CD show some of these features, so they are predisposed to develop SIBO and a complicated clinical course of CD. Bacterial overgrowth or abnormal microflora (dysbiosis) within the gut have been described in the past in CD patients (Manichanh et al., 2006; Lupp et al., 2007; Sartor, 2008), and they are present in the early stages of CD being further amplified by antibiotic treatment (Gevers et al., 2014; Kowalska-Duplaga et al., 2019). Also, healthy first-degree relatives of CD patients show an altered microbiota suggesting a genetic predisposition to develop this condition (Joossens et al., 2011). In addition, there are differences in the diversity of microbiota depending on disease activity: between inflamed areas in different IBD phenotypes, and in microbial metabolism (Forbes et al., 2016; Franzosa et al., 2019; Lloyd-Price et al., 2019). Overall, the dysbiotic profile in CD patients is characterized by a decrease in *Bacteroidetes* and *Firmicutes*, and an increase in *Enterobacteriaceae* microbial populations (Frank et al., 2007; Kostic et al., 2014; Khan et al., 2019). The decrease in *Faecalibacterium prausnitzii* has been widely observed (Hedin et al., 2016), which is a bacterium with an important role in the regulation of Th17 cells (Zhang M. et al., 2019). In fact, this species, together with *Lactobacillus* and *Bifidobacterium*, among others, display a protective role in intestinal inflammation (Hrdý et al., 2020; Singh et al., 2020). Additionally, reduced abundance of *Lachnospiraceae* and *Ruminococcaceae* families producing short-chain fatty acids (SCFAs) are associated with poor response to TNF-α biologic therapy and frequent relapses in CD (Yilmaz et al., 2019). Also, the altered gut microbiome is associated with enrichment of sulfate-reducing bacteria in active CD patients and mice with microbiota from CD patients with active disease (Metwaly et al., 2020).

Decreased complexity and diversity of commensal bacteria that promote intestinal homeostasis play a critical role in CD due to the disrupted capacity of the microbiota to exclude pathogens, which can favor inflammatory responses. *Mycobacterium avium paratuberculosis* (MAP) is an obligate intracellular pathogen detected in intestinal samples of CD patients by different molecular biology and cell culture techniques (Mishina et al., 1996; Schwartz et al., 2000). The contribution of MAP in the pathogenesis of CD was further confirmed by the detection of cultivable MAP in the blood of patients with CD (Naser et al., 2004). Therefore, MAP has been proposed as a potential etiologic infectious agent of CD, although this hypothesis remains to be validated (McNees et al., 2015). Another microbial pathogen such as adherent/invasive *E. coli* has been detected in biological samples of the ileum in patients with CD (Darfeuille-Michaud et al., 2004), and more interestingly, there is an increased severity of CD in those patients with high levels of serum antibodies detecting porin C present in the outer membrane of *E. coli* (Mow et al., 2004). *Clostridiodes difficile* is also an opportunistic pathogen in IBD patients frequently causing symptoms ranging from diarrhea to fulminant colitis and death (D’Aoust et al., 2017). IBD patients have a higher risk of *Clostridiodes difficile* infection, which is associated with longer hospitalization periods and increased resource costs (Nguyen et al., 2008; Razik et al., 2016). Despite literature showing a clear perturbation in IBD microbiota, it is not a clear cause–effect link. It is known that the inflammatory state in CD affects the microbial composition (Craven et al., 2012) but also that IBD microbiota can induce intestinal inflammation (Nagao-Kitamoto et al., 2016). In this regard, treatments addressed to restore healthy gut microbiota in IBD, such as administration of prebiotics and probiotics (Limketkai et al., 2020), antibiotic therapy (Castiglione et al., 2003), and fecal microbiota transplantation (Britton et al., 2020) need further investigations.

Antibiotics used on patients with inflammatory intestinal disease are targeted toward bacteria that, in turn, favor the colonization of intestinal niches by other members of the intestinal microbiota. Relevance of fungal microbiota dysbiosis have been described in patients with CD (Liguori et al., 2016), and antibodies to anti-*Saccharomyces cerevisiae* have been detected in CD (Seow et al., 2009). The interaction between intestinal fungi and host immune system occurs through receptors of the host innate immune system such as Dectin-1 (Iliev et al., 2012). Recently, poor mucosal healing in CD has been associated with overgrowth of *Debaryomyces hansenii* underlying that not only bacteria but also fungi species may modulate the intestinal inflammatory disease (Jain et al., 2021). Relevance of fungi in mucosal healing was evidenced in the study of Jain et al. (2021) by detecting the presence of *D. hansenii* in intestinal wounds with impaired healing after antibiotic treatment, whereas the administration of antifungal amphotericin B reduced fungi detection and increased wound regeneration. Oral gavage of *D. hansenii* altered crypt regeneration in conventional mice not treated with antibiotics and increased the severity of experimental colitis. The authors confirmed that macrophages were recruited in the areas colonized by *D. hansenii* and that CCL5 and type I IFN secreted by myeloid cells are required to alter mucosal healing, supporting CCL5 as a potential target in CD. Additionally, changes in the enteric virome associated with an expansion of *Caudovirales* bacteriophages have been described in patients with CD (Norman et al., 2015). Viral infection by the enteric murine norovirus in experimental models carrying the CD susceptibility gene ATG16L1 is associated with multiple pathologic abnormalities in the intestine (Cadwell et al., 2010). Even if increasing knowledge is required to understand the interactions existing between intestinal microbiota and the host during CD, we can assume that intestinal microbes play an active role in the progression of intestinal inflammatory disease.

The intestinal epithelium acts as a physical and antimicrobial barrier against pathogenic bacteria and environmental antigens (Okamoto and Watanabe, 2016). When the intestinal barrier is disrupted, commensal microbiota, which in physiological conditions exist in a symbiotic relationship with humans, can cross the epithelium and contribute to intestinal inflammation. The intestinal mucosal barrier is composed of both the outer mucus layer, which is comprised by secreted mucinous and antibacterial components, and the inner subepithelial elements involving the immune system (Salim and Söderholm, 2011). Epithelial cells together with M-cells, mucus-secreting globet cells and Paneth cells form a polarized monolayer structure linked by apical junctions which are formed by tight junctions and subadjacent adherens junctions (Turner, 2009). The junctional complex is composed of transmembrane and peripheral proteins including actin, claudins, occludins, zonula occludens (ZO)-1, and junctional adhesion molecules. Enteric glial cells located in the intestinal mucosa also regulate the permeability of the intestinal epithelial barrier in CD by producing 15-hydroxyeicosatetraenoic acid, a polyunsaturated fatty acid that increases the expression of ZO-1 (Pochard et al., 2016). Crucial functions of the intestinal barrier include maintenance of intestinal homeostasis by allowing the absorption of essential nutrients, as well as tolerance to commensal bacteria, and prevention of the entry of injurious bacterial components. A disturbance in one of the components that are involved in the epithelial barrier function can increase its permeability leading to an impaired ability to avoid BT. Altered expression of mucins 1, 3, 4, and 5B in the ileal mucosa of patients with CD favor the binding of microbes to the intestinal surface (Buisine et al., 1999). Additionally, the protein composition of tight and adherens junctions on intestinal cell–cell contacts is altered on CD patients (Zeissig et al., 2007; Kosovac et al., 2010). Disturbances in the permeability of the intestinal barrier associated with a derangement of the tight junction were also probed by freeze–fracture electron microscopic analysis (Marin et al., 1983a,b). Reduced integrity of the intestinal barrier leads to an increased absorption of luminal microbial antigens and serum concentrations of endotoxins, lipopolysaccharide-binding protein (LBP), and CD14s, which are markers of disease activity in CD (Pastor Rojo et al., 2007; Lakatos et al., 2011).

The importance of genetic background as a contributing factor to the impaired barrier function in CD comes from studies with first-degree relatives of patients with CD showing that NOD2 3020insC mutation is associated with increased mucosal permeability (Irvine and Marshall, 2000; Buhner et al., 2006). Also, a gene polymorphism in adherens junction protein E-cadherin (CDH1 gene) was observed in some patients with CD resulting in a cytoplasmic mis-localization of the protein pointing to a defect in the intestinal barrier structure (Muise et al., 2009). From a clinical point of view, increased intestinal permeability has been reported to predict an increased risk of relapse in CD patients on remission (Arnott et al., 2000; Tibble et al., 2000) and is considered as a risk factor for CD onset (Turpin et al., 2020). Serum proteins and antibodies related to immune responses to intestinal microbiota can predict the development of CD up to 5 years before the diagnosis (Torres et al., 2020). Therefore, leaky gut in patients with CD may allow the passage of intestinal microbes across the intestinal epithelium and drive local and systemic proinflammatory responses that worsens the prognosis in patients with CD.

As a consequence of the impaired intestinal integrity, CD patients need to respond to the frequent bacterial challenges to which they are exposed to and ensure the clearance of translocating bacteria. A competent intestinal antimicrobial peptide response is required to protect host from pathogens and to provide tolerance to normal flora (O’Neil et al., 1999; Ramasundara et al., 2009). Several studies have shown that Paneth cells in CD patients display alterations in the production and the activity of different antimicrobial peptides such as cathelicidin (Schauber et al., 2006; Tran et al., 2017), α-defensins (Wehkamp et al., 2005; Elphick et al., 2008), and β-defensins (Kocsis et al., 2008; Schroeder et al., 2011), which are detrimental in the control of BT. Mutations in ATG16L1 and NOD2 in Paneth cells are associated with abnormalities in packaging and secretion of antimicrobials (Liu et al., 2014; VanDussen et al., 2014), therefore, affecting the antibacterial activity of the intestinal barrier by reduced secretion of mucosal α-defensins observed in CD (Wehkamp et al., 2004, 2005; Kobayashi et al., 2005; Petnicki-Ocwieja et al., 2009). Intriguingly, serum levels of α-defensins, but not β-defensins, are increased in patients with CD and they have been associated with serum C-reactive protein and TNF-α (Yamaguchi et al., 2009), while in healthy donors, peripheral α-defensins remain constitutively expressed and β-defensins are induced by bacterial-derived products (Fang et al., 2003). We have demonstrated that bactDNA can modulate the expression of β-defensin (DEFB) 2 and cathelicidin LL-37 through the mediation of NOD2 status by the signaling pathway of nuclear factor (NF)-κB in CD (Gutiérrez et al., 2011). This evidence suggests that the NOD2 gene regulates signaling pathways linked to defensins and cathelicidins through the nuclear factor (NF)-κβ (Wehkamp et al., 2004; Voss et al., 2006). Consequently, patients with a NOD2 mutation have an increased likelihood of developing ileal CD, and it is commonly accepted that an impaired NOD2 function can lead to a poor host clearance of bacteria, which can promote and perpetuate intestinal inflammation. A reduction in bacterial clearance has also been related to polymorphisms in ATG16L1 and IRGM genes, autophagy genes related to CD susceptibility (Hampe et al., 2007; Parkes et al., 2007; Rioux et al., 2007). A mutation on ATG16L1 and IRGM genes induces an injured autophagy pathway, resulting in a defective elimination of damaged cellular organelles and long-lived proteins as well as an altered degradation of intracellular bacteria.

Consequently, increased BT burden and altered microbial clearance in CD patients will induce sustained intestinal inflammatory responses that will be the topic addressed in the following section.

## Inflammatory Response to Bacterial Translocation in Crohn’s Disease

**FIGURE 1:**
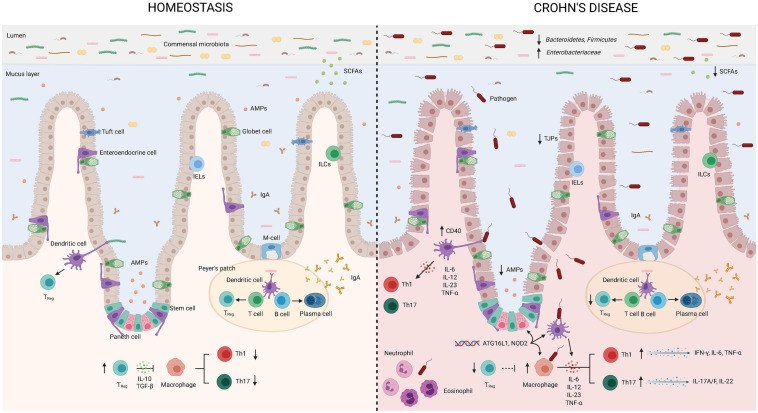
Bacterial translocation in Crohn’s disease. Intestinal tolerogenic mechanisms are altered in Crohn’s disease leading to sustained inflammatory status. Integrity of the epithelial barrier is altered due to reduced expression of tight junction proteins. Increased populations of *Enterobacteriaceae* and pathogens, as well as reduced *Bacteroidetes*, *Firmicutes*, and populations of bacteria producing short chain fatty acids define intestinal dysbiosis in CD. Paneth cells display alterations in the production and secretion of antimicrobial peptides that can be explained by the gene status (NOD2). Translocating bacteria or its products can activate antigen presenting cells as macrophages and dendritic cells. Gene variants in ATG16L1 and NOD2 are associated with abnormalities in the secretion of antimicrobial peptides by Paneth cells and altered function of intestinal DCs and macrophages. Dendritic cells express higher levels of CD40 leading to increased interactions with T-lymphocytes and production of proinflammatory cytokines. Low regulatory T-lymphocyte differentiation will favor Th1 and Th17 subsets that will further produce proinflammatory cytokines. Neutrophils and eosinophils will be recruited to the site of infection and further contribute to induce an inflammatory environment in the attempt to eliminate translocating bacteria. AMPs, antimicrobial peptides; IgA, Immunoglobulin A; IL, interleukin; TGF, transforming growth factor; TNF, tumor necrosis factor; IELs, intraepithelial lymphocytes; ILCs, innate lymphoid cells; SCFAs, short chain fatty acids; TJPs, tight-junction proteins; T_REG_, regulatory T-cells. This figure has been created using the BioRender platform.

The GI tract represents the largest surface area exposed to a wide and heterogeneous community of bacterial antigens. The gut is strictly regulated by innate and adaptive defense mechanisms, which altogether interact with commensal bacteria to promote the maintenance of intestinal homeostasis. Since CD is an immune-mediated condition triggered by environmental factors that imbalance the gut microbiota, perturb the intestinal barrier, and abnormally stimulate the gut immune response, an alteration in any of these compartments determines how the inflammatory immune response develops and may predispose to a disturbance of the bowel, leading to chronic inflammation. Here, we will describe in each one of the components involved in the process of BT and its role in the gut immune response and inflammation, which are summarized in Figure 1.

Intestinal barrier permeability increases the bacterial pressure to which the immune system needs to respond. When BT occurs, the first line of defense against microbial pathogens in the gut is composed of germline-coded pattern-recognition receptors (PRRs), which belong to the innate immune system (Medzhitov and Janeway, 2002). These receptors are located on both the extracellular or the intracellular side, and they recognize molecular patterns that are conserved in bacteria: pathogen-associated molecular patterns (PAMPs). PRRs are composed of transmembrane Toll-like receptors (TLRs), which have a key role in microbial recognition and induction of antimicrobial genes, and cytosolic NOD receptors whose main activity relies on bacterial clearance (Cario, 2005). Bacterial antigens such as endotoxin, forming complexes with LBP or DNA can sense and activate monocytes and macrophages via TLR receptors triggering the release of proinflammatory cytokines and chemokines TNF-α, IL-6, IL-8, IL-21, or IFN-γ through (NF)-κβ pathway (Hemmi et al., 2000; Wagner, 2002), similar to what MDP does via NOD2 (Lala et al., 2003; Eckmann and Karin, 2005) contributing to microbiota dysbiosis and tissue damage.

NLRs are important mediators in the control of intestinal inflammation, since the presence of gene polymorphisms in these molecules confers susceptibility to CD (Cummings et al., 2010). The activation of NLRs by PAMPs or danger-associated molecular patterns (DAMPs) result in downstream NF-kB signaling or caspase-1-mediated formation of inflammasomes (Rubino et al., 2012). NLRP3 inflammasome is activated in CD (Lazaridis et al., 2017), and its inhibition suppress the release of proinflammatory mediators (Liu et al., 2017). However, results from experimental models show controversial results since adverse and protective roles for NLRP3 have been reported. Attenuated colitis was described in both NLRP3-deficient mice (Bauer et al., 2012) and after selective blockade of NLRP3 (Perera et al., 2018) in different animal models of intestinal inflammation, whereas inflammatory progression associated with altered intestinal integrity and increased mortality have also been outlined in NLRP3 knockout mice with experimental colitis (Zaki et al., 2010). It seems that the contribution of NLRP3 to the pathogenesis of IBD is highly influenced by the environment, including intestinal microbiota, as this molecule not only controls potential invading pathogens (Song-Zhao et al., 2014) but also participates in an inflammatory lytic cell death of innate immune cells mediated by caspase-1, known as pyroptosis (Fink and Cookson, 2006). NLRC4 is another relevant member of the NLR family able to detect flagellin and components of the type III bacterial secretory apparatus. NLRC4 inflammasome expressed in intestinal phagocytes seems to become relevant in the discrimination of pathogen and commensal microbiota through the production of IL-1β (Franchi et al., 2012). Additionally, NLRC4-deficient mice were more susceptible to experimental colitis associated with increased mortality following flagellated-*Salmonella* infection (Carvalho et al., 2012). Recent studies demonstrated that NLRP6 inflammasome can be activated either by lipoteichoic acid from *Listeria monocytogenes* (Hara et al., 2018) or via interaction with LPS and ATP (Leng et al., 2020). NLRP6 not only becomes relevant in the host immune response to microbial infections through the production of IL-18 but also mediates the secretion of mucins by globet cells (Wlodarska et al., 2014). Indeed, NLRP6-deficient mice showed more severe experimental colitis associated with a thinner mucus layer, susceptibility to bacterial infections, and altered intestinal microbiota (Elinav et al., 2011; Wlodarska et al., 2014). AIM2 belongs to the innate immune receptors sensing self or foreign cytosolic double-stranded DNA that results in the activation of caspase-1 mediated by the AIM2 inflammasome and consequent release of processed IL-1β and IL-18 (Hornung et al., 2009). AIM2 protects against intestinal inflammation induced by experimental colitis by limiting the growth of *E. coli* and by affecting to the production of antimicrobial peptides (Hu et al., 2015).

In this first-line defense system, macrophages and dendritic cells (DC) play a key role. Alterations in these cell populations have been widely studied in the context of human IBD. Macrophages derived from the peripheral blood monocytes from CD patients showed impaired secretion of cytokines after *E. coli* insult and TLR ligation, contributing to a defective bacterial clearance (Smith et al., 2009). In addition, these cells showed an altered expression of surface markers, abundant secretion of IFN-γ, IL-6, IL-23, and TNF-α (Kamada et al., 2008). TNF-α and IFN-γ are major contributors to intestinal permeability (Cao et al., 2013; Xu et al., 2019). Intestinal DC directly samples luminal bacteria and transfers bacterial antigens to the mesenteric lymph nodes and Peyer’s patches to recruit neutrophils and eosinophils, and to modulate the subsequent T-cell responses (Rescigno et al., 2001; Hart et al., 2005).

Neutrophils recruited to the site of infection phagocyte and kill invading pathogens through reactive oxygen species (ROS) production, neutrophil extracellular traps (NETs), and generation of lytic proteins (Wéra et al., 2016). Neutrophils can also orchestrate local immune responses by releasing cytokines and chemokines such as IL-8, CXCL1, CCL3, CCL4, and CCL20 among others, that can interact and recruit leukocytes from innate and adaptative immune populations including other neutrophils, basophils, eosinophils, macrophages, monocytes, DCs, and T cells (Tecchio and Cassatella, 2016). The proper functioning of neutrophils is crucial to resolve the inflammation induced by translocating pathogens since the lack of function or an increased neutrophil activity may be the origin of intestinal inflammation. While accumulation of neutrophils in the lamina propria correlates with the activity of the disease in UC (Bressenot et al., 2015), several research lines report deficient neutrophil activity in CD. Some studies suggest that neutrophils from CD patients show impaired ROS generation (Hayee et al., 2011); however, it is not clear if there exists an intrinsic failure in neutrophils activity in CD or if this is due to defective macrophage signaling and consequent reduced neutrophil recruitment within the inflammatory area (Segal and Loewi, 1976). Reduced production of the neutrophil chemokine IL-8 support the abnormal neutrophil chemotaxis observed in inflammatory lesions during CD (Marks et al., 2006). The reduced activity of neutrophils against luminal microbes may partially explain the chronic local and systemic inflammation underlying CD induced by a permanent activation of macrophages and T cells (Segal, 2018). On the other hand, eosinophilia is present in CD mucosal biopsies, and it is specially abundant in mucosal nerves (Yantiss, 2015). Some clinical studies suggest peripheral blood eosinophilia as a marker of worse outcome in CD patients (Click et al., 2017).

Under physiological conditions, DC ensures homeostasis inducing a tolerogenic intestinal state (Kretschmer et al., 2005; Tsuji and Kosaka, 2008; Raker et al., 2015). However, the proinflammatory intestinal milieu in CD hinders the tolerogenic profile of these cells (Iliev et al., 2009). During inflammation, there is an increase in the number, maturation, and retention of DC, contributing to inflammation (Middel et al., 2006; Verstege et al., 2008). In CD, DC express higher levels of CD40 leading to increased interactions with T-lymphocytes and the production of great amounts of proinflammatory cytokines (Senhaji et al., 2015) such as IL-6 and IL-12, which are related to microbial changes (Ng et al., 2011) and dysregulation in T-cell apoptosis (Atreya et al., 2000) and, also, IL-8 and TNF-α (Baumgart et al., 2009). TNF-α is the key effector cytokine driving tissue injury during intestinal inflammation (Garrett et al., 2007); it can modulate intestinal mucus secretion and composition (McElroy et al., 2011) and the epithelial barrier function (Al-Sadi et al., 2016; Grabinger et al., 2017). In addition, NOD2 variants, which are most widely detected genetic risk variants associated with CD pathogenesis, disturb DC bacterial sensing, cytokine production, and antigen presentation pathways (Cooney et al., 2010).

Innate lymphoid cells (ILC) cells are also involved in the innate immune response in CD. Its biological relevance lies in their capacity to sense environmental signals and to respond with the secretion of cytokines, producing a profound impact on epithelial cells (Maloy and Powrie, 2011; Sonnenberg and Artis, 2012), and conditioning T-cell responses (von Burg et al., 2015). In CD patients, there is an expansion of an intraepithelial ILC1 subset that produces IFN-γ in response to stimulation with IL-12 and IL-15 (Bernink et al., 2013; Fuchs et al., 2013), possible implication of ILC2 in the development of intestinal fibrosis through IL-13 secretion (Bailey et al., 2012), and ILC3 accumulation in inflamed areas, where they contribute to inflammation through increased IL-17 production and the recruitment of other immune cells (Geremia et al., 2011).

Mucosal CD4^+^ T-cells are central players in maintaining a proinflammatory cytokine response by pushing a predominantly T-helper type 1 (Th1)-mediated inflammatory state in environments where IL-12 is released by antigen-presenting cells (APCs). For many years, it was accepted that CD was mainly mediated by Th1 cells (Brand, 2009), based on the fact that an elevation of the Th1 cytokines was observed in CD patients (Fuss et al., 1996; Monteleone et al., 1997; Parronchi et al., 1997; Pizarro et al., 1999). However, further studies had led to the identification of another subset of CD4^+^ T characterized by the production of IL-17A, IL-17-F, and IL-22, which mediate T-helper type 17 (Th17) cells responses in CD (Strober and Fuss, 2011). An increase in Th17 cytokines produced by Th17 cells in inflamed gut mucosa (Fujino et al., 2003; Nielsen et al., 2003) as well as isolation and characterization of Th17 cells from gut mucosa of patients with CD (Annunziato et al., 2007) has supported the role of this cell population in IBD pathogenesis. CD displays a complex frame where Th1 and Th17 responses shift and depend on disease progression (Friedrich et al., 2019).

The differentiation of naïve T cells to Th17 cells is induced primarily by IL-6 and transforming growth factor (TGF)-β (Bettelli et al., 2006; Ivanov et al., 2006) and further reinforced by IL-1β and IL-23 (Langrish et al., 2005; Chung et al., 2009). IL-23 displays a central role in the maintenance and terminal commitment of naïve cells (Stritesky et al., 2008; McGeachy et al., 2009) and is implicated on the proliferation and expansion of Th17 cell populations (Veldhoen et al., 2006; Bettelli et al., 2007). IL-23R signaling in T cells drives the accumulation of intestinal Th17 cells while reducing the differentiation of tolerogenic FoxP3+ T-cells, as well as a reduced production of IL-10 by T-cells (Ahern et al., 2010). IL-23 induces T-cell expression of IL-17A, IL-17F, TNF-α, and granulocyte macrophage colony-stimulating factor (GM-CSF) (Langrish et al., 2005; Tait Wojno et al., 2019). Increased expression of IL-17A and IL-17F has been detected in the mucosa of patients with active CD (Fujino et al., 2003; Nielsen et al., 2003; Hölttä et al., 2008; Seiderer et al., 2008; Geremia et al., 2011). In addition, genome association analysis has revealed many IL-23R variants linked with CD (Cummings et al., 2007; Cotterill et al., 2010), and some IL-23R loss of function mutations are protective in both UC and CD (Kim et al., 2011). Therapies targeted to IL-23 and its signaling pathways are promising approaches in CD treatment as observed in other inflammatory disorders such as psoriasis or multiple sclerosis (Neurath, 2017; Visvanathan et al., 2018). Antibodies targeting the IL-23 signaling are classified in those recognizing the p40 subunit shared by IL-12 and IL-23 or the p19 subunit unique in IL-23. Ustekinumab is an anti-p40 antibody with a favorable safety profile due to low rate of adverse events that shows a high rate of response and induce remission in moderate to severe CD patients (Feagan et al., 2016). Results from a phase 2 clinical trial in CD patients who failed in anti-TNF-α showed that selective IL-23 blockade using brazikumab, an anti-p19 antibody, was associated with clinical improvement at weeks 8 and 24, and higher serum levels of IL-22 (Sands et al., 2017). Similarly, another anti-IL-23-specific antibody, risankizumab, induced clinical remission in CD patients with active disease at week 12 (Feagan et al., 2017). Biological treatments targeting the IL-17 signaling are effective in psoriasis (Langley et al., 2014). Nevertheless, antibody therapy against IL-17, secukinumab, and its receptor IL-17R, brodalumab, have demonstrated unexpected results in CD, since two different clinical trials reported that the administration of secukinumab and brodalumab in moderate to severe CD patients were not effective and reported more adverse events and worsening of CD (Hueber et al., 2012; Targan et al., 2016).

IL-22 is another Th17-derived cytokine whose implication in IBD has been controversial. Some studies point to a protective role in the intestinal epithelium, stimulating the production of antimicrobial peptides (Okumura and Takeda, 2017), mucus secretion (Sugimoto et al., 2008), intestinal cell proliferation and survival (Zhang X. et al., 2019), and mucosal healing (Patnaude et al., 2021), while others mark that IL-22 may drive intestinal inflammation and gut epithelial cell death (Zha et al., 2019). These data suggest that its role during intestinal inflammation is highly context dependent. In fact, in the presence of eosinophilia, which is common during intestinal inflammation, IL-22 protective actions could be insufficient due to an increase in IL-22-binding protein (IL-22BP) (Martin et al., 2016).

Intensive research aiming to elucidate the contribution of Th17 responses to IBD have reported that IL-17 may exacerbate (Zhang et al., 2006) or protect (Yang et al., 2008; O’Connor et al., 2009) against intestinal inflammation depending on the experimental model studied. Results from Zhang and colleagues showed that IL-17R knockout mice presented reduced activity of experimental colitis induced by trinitrobenzenesulfonic (TNBS) acid. In line with this, the treatment with a soluble IL-17 receptor IgG fusion lessened intestinal inflammation induced by TNBS. On the other hand, studies conducted in animal models of colitis induced either by dextran sulfate sodium in IL-17 knockout mice or by CD45RB^hi^ adoptive transfer using IL-17 or IL-17R-genetically deficient T-cells revealed an accelerated disease, therefore suggesting a protective role of IL-17 in those experimental systems. In order to determine the contribution of both Th1 and Th17 responses in CD, Sakuraba et al. isolated dendritic cells and lymphocytes from mesenteric lymph nodes of patients with CD. The authors observed that isolated CD4+ T-cells were producing increased levels of IFN-γ and IL-17, but isolated dendritic cells were activating CD4+T-lymphocytes toward the production of IFN-γ (Sakuraba et al., 2009). Taken together, these evidences suggest that BT might contribute to modulate the inflammatory response in CD via enhancing a Th1/Th17 response associated with the presence of bacteria or their products, which perpetuates the progression of the disease in a subgroup of patients.

The intestinal Treg population is relevant in the inflammatory responses to BT in CD, as they oversee tissue repair and immunological tolerance toward food antigens and microbiota in the gut, contributing to intestinal homeostasis (Kim et al., 2016; Tanoue et al., 2016; Xu et al., 2018). They belong to CD4+ lymphocytes and can suppress the immune response interacting with different components of the innate and adaptive immune response. Treg cells are highly heterogenous and express different lineage-specific transcription factors and cellular markers in different scenarios (Zhang et al., 2020). Treg cell populations produce IL-10 and TGF-β, and they can be naturally synthesized through thymic selection or induced after antigenic stimulation outside the thymus (Roncarolo et al., 2006; Sakaguchi et al., 2010), also in the gut by mucosal CD103^+^ dendritic cells via a TGF-β and retinoic acid-dependent mechanism (Coombes et al., 2007). Treg secretion of IL-10 is important to control the gut balance. In fact, intestinal Th1-mediated inflammatory responses result in spontaneous colitis in IL-10-deficient mice (Davidson et al., 1996), and polymorphisms in the human IL-10R result in exacerbated intestinal immune responses (Glocker et al., 2009).

Changes in the percentage of Treg cells in patients with IBD have been reported (Maul et al., 2005), and a decreased number of CD4+ CD25+ FoxP3+ Treg cells have been observed in the lamina propria of patients with CD-related NOD2 variants (Rahman et al., 2010). Also, mutations in FOXP3 gene are related to the development of IBD (Okou et al., 2014). In the inflammatory milieu of CD, some groups have reported an enhanced recruitment of Treg cells in mucosal areas, suggesting a deficient suppressive activity during inflammation (Chamouard et al., 2009). These could be explained through changes in its cytokine profile similar to Th17 cells in the context of IBD (Hovhannisyan et al., 2011; Ueno et al., 2013) and also a diminished expression of transcription factors involved in Treg regulation in CD (Qiao et al., 2013). On the other hand, recently, a subset of Treg CD161+ cells has been found highly enriched in the mucosa of CD patients, which are involved in wound healing and associated with reduced inflammation (Povoleri et al., 2018). All of these suggest that different Treg subsets could behave differentially in IBD. Due to its immunomodulatory capacity, therapies targeting this cell population are being assessed with promising results (Desreumaux et al., 2012; Trotta et al., 2018; Clough et al., 2020).

The microbiome is key in the equilibrium between Treg and Th17 in the gut (Lochner et al., 2011; Ohnmacht et al., 2015; Sefik et al., 2015). Microbiota from IBD donors into germ-free mice reduced the presence of RORγt+ Treg cells but increased Th17 and Th2 populations (Britton et al., 2019). On the other hand, commensal microbiota can promote CD4+ CD25+ FoxP3+ Treg cells *in vivo*, which control the innate inflammatory cascade to translocating microbes by reducing proinflammatory cytokine production, reducing T-cell proliferation, reducing dendritic cell co-stimulatory molecule expression, and attenuating (NF)-κβ activation (O’Mahony et al., 2008). SCFAs produced by *Bifidobacteria* and *Clostridia*, like butyrate, cause inhibition of histone deacetylase (HDAC), promoting FoxP3 expression (Arpaia et al., 2013) and production of retinoic acid (Smith et al., 2013; Schilderink et al., 2016) polysaccharide A of *Bacteroides fragilis* induces an intestinal tolerogenic environment by promoting the IL-10-producing Foxp3+ Treg population (Round and Mazmanian, 2010), and indole-3-aldehyde, produced by *Lactobacillus reuteri*, is a tryptophan precursor involved in the plasticity of T cells (Lamas et al., 2016). In addition, *Clostridiodes* spp. mixture transplantation is also associated with increased counts of intestinal Treg cells in mice (Atarashi et al., 2013; Narushima et al., 2014).

## Efficacy of Anti-TNF-α Treatment in Patients With Bacterial Translocation

Biologic treatments including anti-TNF-α, adhesion molecule inhibitors, and p-40 IL-12/23 inhibitor, ustekinumab, are effective therapies for patients with moderate to severe IBD (Katsanos et al., 2019). Anti-TNF-α monoclonal antibodies were the first biologic agents that demonstrated effectiveness in the treatment of CD (Rutgeerts et al., 1999; Hanauer et al., 2002; Sands et al., 2004; Xiao et al., 2016) as TNF-α is increased in the intestinal mucosa of IBD patients (Breese et al., 1994; Dionne et al., 1997). Increased intestinal TNF-α could be directly involved in BT, as it can disrupt intestinal epithelial integrity (Al-Sadi et al., 2016; Grabinger et al., 2017) and mediate tissue injury (Garrett et al., 2007). However, it is known that 30–40% of patients with IBD under anti-TNF therapy show a primary non-response, and up to 50% may present adverse events or develop secondary non-response over time (Ben-Horin and Chowers, 2011; Papamichael et al., 2017). Focusing on the efficacy of anti-TNF-α therapy, further research has also found that undetectable serum through concentration of anti-TNF-α levels (Maser et al., 2006) and decreased free TNF-α binding capacity of anti-TNF-α drugs (Ainsworth et al., 2008) are predictors of poor response to anti-TNF-α treatment of patients with CD. Even if serum levels of TNF-α have also been proposed to predict the efficacy of anti-TNF-α in CD patients (Martínez-Borra et al., 2002), several studies have reported that serum TNF-α is not a good predictor of clinical response to anti-TNF-α therapy (Ogawa et al., 2012). Besides clinical factors and the development of antibodies against anti-TNF-α agents (Baert et al., 2003), several other factors such as BT and a susceptible genotype, intestinal dysbiosis, and even the Treg population may have a role in this loss of response.

In the past, we investigated the effects of different gene variants and BT in the efficacy of anti-TNF-α therapy in CD. We identified a subgroup of CD patients characterized by the presence of a variant NOD2 genotype, in combination or not with a variant ATG16L1 genotype, who may need an intensified anti-TNF-α drug schedule since they showed increased bactDNA translocation, augmented inflammatory response, and increased risk of relapse. In detail, the presence of a variant NOD2 genotype, either alone or combined with ATG16L1 variant genotype, was associated with increased bactDNA translocation, and the presence of serum bactDNA was associated with relapse at 6 months. Patients with bactDNA showed increased proinflammatory cytokines response that was further augmented in patients who were also carrying combined NOD2/ATG16L1 variants. A variant NOD2 genotype correlated with reduced phagocytic and bactericidal activities in neutrophils and exacerbated *in vitro* TNF-α secretion in response to *E. coli*, suggesting that neutrophils from CD patients carrying a variant NOD2 genotype have altered bacterial clearance. Evaluation on anti-TNF-α therapy on patients carrying NOD2/ATG16L1 combined genotypes revealed that most of these patients were on an intensified anti-TNF-α drug schedule. Moreover, free anti-TNF-α levels were significantly decreased in the serum of patients with bactDNA translocation and a variant NOD2 genotype and, especially, in patients with a combined NOD2/ATG16L1 variant, suggesting that increased drug consumption is necessary on these patients to promote an adequate tolerogenic response (Gutiérrez et al., 2014). We further demonstrated that the presence of bactDNA in CD patients is a significant independent risk factor of short-term relapse in those in remission, especially in the ones with mucosal lesions, suggesting that the presence of mucosal damage is not essential for BT, but it contributes to it, in synergy with bactDNA (Gutiérrez et al., 2016). In line with this, we observed that the increase in bactDNA and TNF-α in CD patients could be related with a variant in IL-26 gene. This variant was associated with an impaired antibacterial clearance, increased inflammatory cytokines, and an increment in anti-TNF-α consumption in CD patients (Piñero et al., 2017). This also contributes to explain why SNPs in IL-26 gene confer genetic susceptibility to CD (Silverberg et al., 2009). All these findings suggest that BT aggravates the inflammatory response and predisposes to risk of relapse and need of intensified anti-TNF-α drug therapies in susceptible CD patients.

It is well-known that levels of anti-TNF-α determine the treatment response (Moore et al., 2016), but recent studies manifest that intestinal dysbiosis might also play a role in the efficacy of the biologic therapy. Therefore, initial gut microbial composition and cytokine profile before anti-TNF-α therapy, as well as anti-TNF-α-induced microbial changes during the treatment are key in the achievement of clinical remission (Jones-Hall and Nakatsu, 2016; Franzin et al., 2021) and IBD patients with greater gut dysbiosis achieve clinical remission later (Aden et al., 2019). The treatment with anti-TNF-α improves the intestinal dysbiosis in CD by increasing SCFAs producing bacteria like *Anaerostipes*, *Blautia*, *Coprococcus*, *Faecalibacterium*, *Lachnospira*, and *Roseburia* (Kowalska-Duplaga et al., 2020; Seong et al., 2020) and decreasing bacterial species associated with mucosal damage (Busquets et al., 2015; Ribaldone et al., 2019). The relevance of intestinal microbiota in the efficacy of current IBD treatments is certainly an open research field that deserves more in-depth investigations.

Finally, the Treg population has been shown to actively participate in the loss of response to anti-TNF-α. An increased peripheral blood Treg cell population after anti-TNF-α therapy administration is related with increased serum levels of TGF-β and IL-10 and with the clinical improvement observed in patients with CD (Di Sabatino et al., 2010; Guidi et al., 2013). Indeed, we have also reported that Treg population is susceptible to significantly increase after anti-TNF-α administration in CD patients bearing a wild-type NOD2 genotype. Nevertheless, CD patients carrying a polymorphism in NOD2 have lower available serum levels of anti-TNF-α and an impaired capacity to induce the Treg population. Altogether, these results suggest an impaired immunological function in this subgroup of CD patients, as demonstrated by increased serum levels of TNF-α. Accordingly, most of these patients were on anti-TNF-α intensified therapy and showed a more aggressive CD phenotype. Furthermore, we found that CD patients showing perianal lesions had lower circulating Treg population. Thus, immunophenotyping Treg cells in blood of patients with CD can be a fast and helpful methodology to anticipate not only the clinical response to biological therapy but also a more aggressive phenotype of CD (Juanola et al., 2014).

## Future Directions

To predict CD behavior is a topic of strong interest that would greatly improve the welfare of patients. The multifactorial etiology of the disease makes it necessary to consider several aspects from genetic to environmental factors in an attempt to determine the risk of relapse (Timmer et al., 1998; Tibble et al., 2000; Beaugerie et al., 2006; Takeuchi et al., 2006). However, the clinical value, so far, is limited due to lack of specificity.

As shown in this review, many lines of evidence point to the translocation of bacterial products as an important player leading to uncontrolled inflammation in CD patients. Even if the question still arises about BT as the cause or the consequence of intestinal inflammation, it is widely accepted that host–bacterial interactions influence CD. Therefore, evaluating the presence of gut bacterial antigens at a systemic level may constitute a new marker for increased risk of relapse among CD patients. Of particular interest is the combination of BT and CD-related susceptibility genes such as NOD2, which probably facilitates the translocation of bacterial antigens; this is worth exploring in the context of response to TNF-α antagonists and risk of relapse.

Further studies aimed at understanding the interaction between the immune system, both at systemic and mucosal level, gut microbiota, and genetic predisposition will help clinicians to better control and individually treat CD patients in the future.

## Author Contributions

All authors listed have made a substantial, direct and intellectual contribution to the work, and approved it for publication.

## Conflict of Interest

AG has served as speaker, consultant, or advisory member for, or received research funding from MSD, ABBVIE, TAKEDA, KERN PHARMA, PFIZER, OTSUKA, SHIRE, and JANSSEN, outside the submitted work. The remaining authors declare that the research was conducted in the absence of any commercial or financial relationships that could be construed as a potential conflict of interest.

## Publisher’s Note

All claims expressed in this article are solely those of the authors and do not necessarily represent those of their affiliated organizations, or those of the publisher, the editors and the reviewers. Any product that may be evaluated in this article, or claim that may be made by its manufacturer, is not guaranteed or endorsed by the publisher.
